# Editorial: Interactions of the Nervous System With Bacteria

**DOI:** 10.3389/fnins.2021.682744

**Published:** 2021-04-23

**Authors:** Elisa L. Hill-Yardin, Andreas M. Grabrucker, Ashley E. Franks, Ruth Ann Luna, Mastura Monif

**Affiliations:** ^1^School of Health and Biomedical Sciences, Science Technology Engineering Mathematics College, Royal Melbourne Institute of Technology University, Bundoora, VIC, Australia; ^2^Department of Anatomy and Physiology, The University of Melbourne, Melbourne, VIC, Australia; ^3^Department of Biological Sciences, University of Limerick, Limerick, Ireland; ^4^Health Research Institute, University of Limerick, Limerick, Ireland; ^5^Bernal Institute, University of Limerick, Limerick, Ireland; ^6^School of Life Sciences, La Trobe University, Bundoora, VIC, Australia; ^7^Texas Children's Microbiome Center, Texas Children's Hospital, Houston, TX, United States; ^8^Department of Pathology & Immunology, Baylor College of Medicine, Houston, TX, United States; ^9^Department of Neuroscience, Monash University, Melbourne, VIC, Australia

**Keywords:** microbes, gastrointestinal tract, enteric nervous system, brain, neurological disorders, neuroinflammation, diet, dysbiosis

Recent evidence that microbes influence mood and behavior via the gut-brain axis has opened up new avenues for research into neurological disorders. Hence, many studies now employ multidisciplinary approaches assessing for changes in microbial diversity, neuroinflammation as well as alterations in neuronal circuitry that impact brain function in health and disease. Such collaborative research was virtually unheard of in previous decades but holds remarkable promise for identifying novel pathways and therapeutic targets within the gastrointestinal tract to treat brain disorders. This editorial highlights these exciting developments in neuroscience, microbiology, and immunological research by examining 13 articles focused on how the nervous system interacts with bacteria in preclinical and clinical settings. A common theme is the dissection of complex interactions between the nervous system and bacteria as well as the resulting influences on inflammatory pathways, symptoms, or behavior in patient studies and mouse models. Specifically, neuronal-microbial interactions in the context of nervous system disorders ranging from autism, Attention Deficit Hyperactivity Disorder, Alzheimer's Disease and Major Depressive Disorder to migraine and epilepsy are investigated. Overall, we propose that via leveraging our understanding of the gut-brain axis, the modulation of gut microbes leading to significant benefits for brain health can become a reality.

The recent years have seen substantial progress in the understanding of gut-brain interactions. Today, evidence is mounting that the microbiota-gut-brain axis is a key contributor to healthy brain development and function. Accordingly, gastrointestinal (GI) problems and microbial dysbiosis have been linked to several neurological and neuroinflammatory disorders. Consequently, targeting gut microbiota composition to regulate peripheral and central inflammation could serve as means of developing novel treatments or disease modifying strategies for several key neuroinflammatory conditions. This is a rapidly emerging field of neuroscience research and is highlighted in the current issue.

This Research Topic is dedicated to understanding the influence of microbes on brain health. Alterations in the gut microbiota may affect gut-brain signaling via neuronal, endocrine and immunological mechanisms, thereby influencing a range of neuronal network activities and ultimately host behaviors. Zhao et al. showed that emotional behavior is among those behaviors affected. Antibiotic-treated mice with a lower richness and diversity of microbiota display increased anxiety-like, depression-like, and aggressive behaviors (Zhao et al.). These effects on behavior may be caused by a dysregulation of the immune and endocrine system. In line with this, a link between altered microbiota composition and immune system abnormalities was also reported by Sauer and Grabrucker. In their study, a deficiency in the essential trace metal, zinc, changed the composition of the microbiota. Of interest, zinc deficiency has been linked to depression-like behavior in rodents. In this study, the microbial changes were accompanied by increased inflammatory cytokine levels. Furthermore, this study reported abnormal GI morphology and increased GI permeability in these mice, hinting that an increase in intestinal barrier permeability may mediate pro-inflammatory processes (Sauer and Grabrucker).

Relevant to the findings of Sauer and Grabrucker, the role of microbes in modifying the permeability of the mucosal epithelium lining the GI tract was discussed in a review of microbiome-derived neurotoxins by Lukiw. How microbiota might impact the properties of the mucus membrane in the GI tract could also be important in brain disorders. Lukiw discussed the role of the zinc metalloproteinase (also known as *B. fragilis Toxin; BFT* or *fragilysin*), which is released by *Bacteroides fragilis* microbes. *Fragilysin* cleaves tight junction components such as cadherins which can alter the permeability of mucosal and blood-brain barriers. This action would also alter neuro-immune interactions and is age-related and progressive. An important part of the epithelial barrier of the GI tract is the mucus biofilm, which provides a supportive environment for specific microbial populations. Because patients with many neurological diseases have GI issues and microbial dysbiosis, Herath et al., reviewed the evidence for mucus as a potential treatment target in the context of neurological disease (Herath et al.). This review outlined a range of pathways by which gene mutations associated with brain disorders could influence the GI tract's mucus and its microbial populations.

Sharna and colleagues investigated neuro-immune interactions in a mouse model of autism expressing a gene mutation in the nervous system (Sharna et al.). Surprisingly, the weight of the caecum (the equivalent of the human appendix) was reduced in these mice. When analyzing the enteric nervous system in the caecum, mutant mice had more neurons overall and a higher proportion of neurons synthesizing the major inhibitory neurotransmitter (nitric oxide) of the gut. In the same study, Sharna and coauthors found changes in the gut immune system. In these mice, macrophages were labeled with Iba-1 (a marker of microglia in the brain) in the gut-associated lymphoid tissue of the caecal patch. Mutant mice had more Iba-1 labeled macrophages, and these macrophages were smaller and rounder in shape. Interestingly, this could indicate increased immune activity in the autism model. More broadly, these findings suggest that communication between the nervous system and immune cells could be altered in neurological disorders such as autism, and that the gut microbiota plays a key role in regulating such interactions.

Further evidence of the nervous system's interactions and inflammatory pathways in brain disorders include a role for lipopolysaccharide (LPS), lifestyle factors, and both the oral and GI microbiome in Alzheimer's Disease. The accumulation of gut bacteria-derived LPS in neurons is associated with Alzheimer's disease. Lukiw et al. report that LPS entry into human neurons is facilitated by amyloid beta-42 (Aβ42) and discuss the associated pathogenic role of LPS in neurons of Alzheimer's disease brains (Lukiw et al.). The encapsulation of neurons by LPS reduces the release of mRNA from the neuronal nuclei, likely via the interaction of Aβ42 with nuclear pore complexes. Therefore, manipulating the gut microbiome by diet and pre/probiotics to reduce neurotoxic effects within the CNS is an exciting avenue for future research. In line with these findings, Askarova and colleagues brought together a wide range of research to link the brain-gut-microbiota axis and Alzheimer's Disease (Askarova et al.). These authors noted that several lifestyle factors linked to Alzheimer's disease also drive microbiome changes, such as dietary habits, sedentary behavior, and disturbances in circadian rhythms. Changes in the oral microbiome in addition to chronic periodontitis have also been associated with Alzheimer's Disease (Paganini-Hill et al., 2012; Harding et al., 2017; Liu et al., 2019; Panza et al., 2019; Olsen and Singhrao, 2020). Given the potential for factors within the gastrointestinal tract to contribute to the development of neuroinflammation and neurodegeneration, further studies are warranted.

Gut microbiota profiles may contribute to behavioral symptoms associated with a range of neurological disorders, including attention-deficit/hyperactivity disorder (ADHD) and major depressive disorder (MDD). Wan et al. investigated the gut microbiota in children with ADHD. Their results of a case-control study reveal that specific bacteria are significantly increased or decreased in children with ADHD and align with alterations in neurotransmitter metabolic pathways (Wan et al.). However, as Isaiah et al. point out, in addition to the gut signaling to the brain, the reverse (i.e., brain-to-gut signaling) may occur as well (Isaiah et al.), a phenomenon that is currently understudied in the context of ADHD. Yong et al. discussed the role of the microbiota-gut-brain axis, stress, and lifestyle factors for major depressive disorder (MDD) (Yong et al.). The antidepressive effects of probiotics and potential biological mechanisms (including the production of metabolites) to benefit gut health were compared in clinical and animal studies. In addition, Westfall and Pasinetti discussed the role of dietary polyphenols as a possible diseases-modifying treatment for depression (Westfall and Pasinetti). Their review highlighted that synbiotics that combine probiotics with dietary polyphenols such as those found in fruits, tea, herbs, cereal, or wine might be a novel therapy for MDD. This may occur via modulation of multiple metabolic pathways, including the breakdown of kynurenic acid and tryptophan (with influences on glutamatergic activity and microglial function in the brain), serotonergic mechanisms, and immune pathways, including via interferon-gamma and inflammasome activation. Importantly, these authors highlight that the heterogeneity of MDD must be taken into consideration when evaluating the potential therapeutic effects of probiotics.

Migraine is a common, recurrent, and disabling neurological disorder that is associated with alterations in the neurovascular and immunological system. The changes in the nervous system activity that occur in the setting of migraine are commonly associated with gut disorders such as irritable bowel syndrome and inflammatory bowel disease. Chen and others examined the potential for interactions between the nervous system and bacteria in their study of fecal samples from 108 elderly women with and without migraine, which revealed a significant decrease in species diversity and metabolic functions in the gut microbiota of migraine sufferers. These findings suggest that monitoring harmful bacteria such as Clostridium could help with alteration of migraine frequency and potentially even prevention of disease (Chen et al.). Future randomized control trials that assess the efficacy of controlled delivery of “safer” and “less inflammatory” microbiota as a means of controlling migraine frequency are warranted.

Epilepsy is another neurological disorder for which a role for the microbiome is understudied. The ketogenic diet is commonly used to treat medically refractive seizures and epilepsy. Importantly the anti-seizure effects of the ketogenic diet are thought to be mediated by the gut microbiome (Olson et al., 2018). Altering the gut bacteria is thought to regulate seizure frequency and modify disease severity. Rubin and Glazer outlined a potential role for subclinical infection with Bordetella pertussis (BP) in epilepsy (Rubin and Glazer). This work highlighted that cases of subclinical infection are vastly more prevalent than reported pertussis cases and describe incidences of epilepsy occurring after BP infection. Further research into the relationship between epilepsy and BP infection, including BP screening, medical history, and pertussis vaccination is required to assess for an association between BP and seizures.

In summary, this Research Topic brings together evidence for microbial influences in a range of neurological, neuropsychiatric and neurodegenerative disorders including autism, Alzheimer's Disease, Major Depressive Disorder, migraine, and epilepsy. Gut microbiota are thought to influence not only local immunological processes, but also exert effects on the CNS through the disruption of the gastrointestinal mucosal barrier and BBB. The future of gut-brain research holds promise for identifying novel therapeutic approaches to treat many disabling CNS conditions traditionally considered to be associated with dysfunction of the CNS itself. Further research into how modifying gut microbes influence processes in the brain has enormous potential.

## Author Contributions

All authors listed have made a substantial, direct and intellectual contribution to the work, and approved it for publication.

## Conflict of Interest

MM has served on an advisory board for Merck and received speaker honoraria from Merck and Biogen. Her institution receives funding from Merck, Australian National Health Medical Research Council, Brain Foundation, Charles and Sylvia Viertel Foundation, and MS Research Australia. The remaining authors declare that the research was conducted in the absence of any commercial or financial relationships that could be construed as a potential conflict of interest.
